# Epidemiological surveillance of common respiratory viruses in patients with suspected COVID-19 in Southwest China

**DOI:** 10.1186/s12879-020-05392-x

**Published:** 2020-09-21

**Authors:** Yanjun Si, Zhenzhen Zhao, Rong Chen, Huiyu Zhong, Tangyuheng Liu, Minjin Wang, Xingbo Song, Weimin Li, Binwu Ying

**Affiliations:** 1 Department of Laboratory Medicine, West China Hospital, Sichuan University, 37 Guoxue Alley in the wuhou District, Chengdu, Sichuan China; 2 Department of Clinical Laboratory, The First People’s Hospital of Shuangliu District, Chengdu/ West China (Airport) Hospital Sichuan University, Chengdu, Sichuan China; 3 Department of Respiratory and Critical Care Medicine, West China Hospital, Sichuan University, 37 Guoxue Alley in the wuhou District, Chengdu, Sichuan China

**Keywords:** COVID-19, Respiratory viral pathogens, Surveillance research

## Abstract

**Background:**

The outbreak of coronavirus disease 2019 (COVID-19) caused by the severe acute respiratory syndrome coronavirus 2 (SARS-CoV-2) is currently the peak season of common respiratory viral infections. However, the clinical symptoms of most SARS-CoV-2 infected patients are not significantly different from those of common respiratory viral infections. Therefore, knowing the epidemiological patterns of common respiratory viruses may be valuable to improve the diagnostic and therapeutic efficacy of patients with suspected COVID-19, especially in Southwest China (a mild epidemic area).

**Methods:**

A total of 2188 patients with clinically suspected of COVID-19 in Southwest China were recruited from January 21 to February 29, 2020. Nasopharyngeal swabs, throat swabs and sputum specimens were collected to detect SARS-CoV-2 by using real-time reverse transcription-polymerase chain reaction (RT-PCR) and other 12 viruses via PCR fragment analysis combined with capillary electrophoresis. Clinical characteristics and laboratory test findings were acquired from electronic medical records. All data were analyzed to unravel the epidemiological patterns.

**Results:**

Only 1.1% (24/2188) patients with suspected COVID-19 were eventually confirmed to have SARS-CoV-2 infection, and the most frequently observed symptoms were fever (75.0%, 18/24) and cough (20.8%, 5/24). The overall detection rate of other respiratory pathogens was 10.3% (226/2188). Among them, human rhinovirus (3.2%, 71/2188), human parainfluenza viruses (1.6%, 35/2188), influenza B virus (1.2%, 26/2188) and mycoplasma pneumonia (1.2%, 26/2188) were the predominantly detected pathogens in this study. Moreover, the co-infection was observed in 22 specimens. Notably, one COVID-19 case had a coexisting infection with human parainfluenza virus (4.2%, 1/24) and bocavirus was the most common virus tending to occur in co-infection with other respiratory pathogens.

**Conclusions:**

This study reveals the epidemiological features of common respiratory viruses and their clinical impact during the ongoing outbreak of COVID-19 in a mild epidemic area. The findings highlight the importance of understanding the transmission patterns of the common respiratory virus in COVID-19 regions, which can provide information support for the development of appropriate treatment plans and health policies, while eliminating unnecessary fear and tension.

## Background

Since its outbreak in Wuhan, China in December 2019, the severe acute respiratory syndrome coronavirus 2 (SARS-CoV-2)-infected disease (COVID-19) has spread throughout China and continuously to many other countries and regions around the world [1, 2], thus becoming a major public health threat and economic burden worldwide. The research on the clinical characteristics of COVID-19 in China has shown that the most common clinical symptoms of COVID-19 are fever (87.9%) and cough (67.7%), followed by fatigue (38.1%), sputum production (33.4%), shortness of breath (18.6%), muscle pain or joint pain (14.8%), sore throat (13.9%), headache (13.6%), chills (11.8%) and other atypical symptoms. However, these clinical symptoms at the onset of COVID-19 are not different from common respiratory viral infections [3]. Indeed, the patients with acute respiratory infections (ARIs) caused by common respiratory viruses often manifest fever, cough, rhinitis, sore throat, headache and imaging features of pneumonia, which are similar to those of COVID-19 patients, thus leading to seasonal epidemics with pneumonia, bronchitis, severe respiratory failure, and even death [4, 5]. These similar symptoms of ARIs and COVID-19 can hamper the diagnostic and therapeutic efficacy of patients with suspected COVID-19, particularly during the epidemic period of SARS-CoV-2. Therefore, identification of the causative respiratory pathogens is of great importance for stopping the epidemic spread of COVID-19 and contributing to the reduced duration of patient isolation, especially for those who are infected with other common respiratory viruses.

At present, the epidemic situation of COVID-19 is varied across regions. As of February 29, 2020, the National Health Commission has reported a total of 79,824 confirmed COVID-19 cases and 2870 death in Mainland China [6]. There are 143 confirmed cases found in Chengdu city, Sichuan province, southwest of China, and three of them have died [7]. Apparently, this number is much lower than that reported in Wuhan [6]. In the meantime, ARIs are the major causes of outpatient visits and hospitalization in all age groups, especially during winter and spring [8]. A surveillance study on the epidemiology of ARIs among hospitalized young children in Chengdu from 2009 to 2014 reported that 23.0% of ARI patients were identified with human rhinovirus (HRV), 22.7% patients with respiratory syncytial virus (RSV), 13.4% patients with human parainfluenza virus (HPIV) and 8.4% patients with human bocavirus (HBoV) [9]. Furthermore, a systematic review of the causes of ARIs demonstrated that RSV was the most frequently detected pathogen among patients of different age groups [10]. Knowing the epidemiological patterns of common respiratory viral infections at the population scale may help in guiding optimal diagnosis and treatment strategy for ARI patients, and it is more important for areas with relatively lower rates of COVID-19, including Chengdu city. However, most epidemiological studies conducted to date have rarely focused on the distribution patterns of respiratory viral infections and their clinical impact during the ongoing outbreak of COVID-19. A deeper understanding of the actual situation of respiratory viral infections in this location can provide information support for the development of appropriate treatment plans and health policies, while eliminating unnecessary fear and tension. Therefore, in this study, we aimed to investigate the epidemiological, clinical and virologic characteristics of patients with suspected COVID-19, and clarify the actual infection rate and epidemic situation of SARS-CoV-2 and other respiratory pathogens outside the main epidemic area of COVID-19, particularly in Southwest China.

## Method

### Study design and patients

This was a retrospective research investigated on the epidemic situation of SARS-CoV-2 and other viruses in the West China Hospital of Sichuan University, Chengdu, China (a region with mild morbidity of COVID-19), from 21 January to 29 February 2020. The eligibility criteria for the patients enrolled in this study were as follows: (i) fever (≥37.0 °C) and/or respiratory symptoms (e.g., cough, sputum production, hemoptysis, shortness of breath, wheezing, and chest pain, etc.); (ii) with no other underlying pulmonary diseases; and (iii) underwent screening test for 13 respiratory viral pathogens, including SARS-CoV-2. The detailed information, such as clinical manifestations, diagnosis and laboratory test findings, were recorded in case report forms. Patients’ demographics and baseline characteristics were also collected. This study was approved by the Biomedical Research Ethics Committee of West China Hospital (ChiCTR2000030542), and written informed consent was obtained from all participants or their parents/caregivers before sample collection.

### Viral RNA isolation and sample processing

Nasopharyngeal swabs, throat swabs and sputum specimens were collected from patients with clinically suspected COVID-19. After sampling, the specimens were placed into collection tubes filled with a 2 mL virus preservation solution. Total viral RNA was extracted using a QIAamp viral RNA mini kit (Qiagen, Germany). Briefly, 200 μL of each specimen was transferred into a tube containing 210 μL lysis solution and 20 μL proteinase K, followed by incubation at 56 °C for 30 min with vigorous shaking. The obtained cell lysates were transferred to a collection tube and then added with a washing buffer. After centrifugation, the bound RNA extracts were eluted to a final volume of 50 μL.

### SARS-CoV-2 real-time quantitative PCR

Real-time reverse transcription-polymerase chain reaction (RT-PCR) was performed by amplifying two target genes, including open reading frame 1ab (ORF1ab) and nucleocapsid protein (N).

Target 1 (ORF1ab):

Forward primer: CCCTGTGGGTTTTACACTTAA,

Reverse primer: ACGATTGTGCATCAGCTGA,

Fluorescent probe (P): 5′-FAM-CCGTCTGCGGTATGTGGAAAGGTTATGG-BHQ1–3′;

Target two (N):

Forward primer: GGGGAACTTCTCCTGCTAGAAT,

Reverse primer: CAGACATTTTGCTCTCAAGCTG,

Fluorescent probe (P): 5′-FAM-TTGCTGCTGCTTGACAGATT-TAMRA-3′.

Real-time RT-PCR assays were performed on an ABI 7500 system (Applied Biosystems instruments, USA) using a RT-PCR kit (Sansure Biotech Inc., China). Each 30 μL reaction mixture contained 18.5 μL of one-step RT-PCR reaction buffer, 1.5 μL of enzyme mix and 10 μL of RNA as a template. Thermal cycling was initiated at 50 °C for 20 min (for reverse transcription), followed by 95 °C for 10 min and 40 cycles of 95 °C for 15 s and 60 °C for 30 s using the CFX96 Touch RT-PCR system (BIO-RAD Laboratories).

### The multiplex platform for respiratory pathogen panel testing

The nucleic acids of each sample were subjected to multiplex amplification using a Respiratory Pathogen Multiplex Detection Kit (Ningbo Health Gene Technology, Ningbo, China) on an ABI Verity 96 Thermal Cycler (Thermo Fisher Scientific, Carlsbad, CA, USA). The PCR product was then subjected to capillary electrophoresis and fragment analysis using a 3500Dx Genetic Analyzer (Thermo Fisher Scientific, Carlsbad, CA, USA) according to the manufacturer’s protocol. The panel was specifically designed to detect 12 pathogens, namely, influenza A (H1N1) 2009 virus (09H1N1), seasonal H3N2 virus (H3N2), influenza B virus (InfB), human adenovirus (HADV), HBoV, human coronavirus (HCOV; 229E, OC43, NL63, HKU1), human metapneumovirus (HMPV), HPIV, HRV, human respiratory syncytial virus (HRSV), mycoplasma pneumonia (Mp) and chlamydia (Ch), within 4 h.

### Statistical analyses

Kolmogorov-Smirnov test was used to assess the normality of the measurement data. The continuous variables with normal distribution were presented as mean ± standard deviation (SD), while the non-normally distributed variables were expressed as median (interquartile range, IQR). Pearson’s chi-square test, Mann-Whitney U test and Student’s t-test were used whenever appropriate. The categorical variables were expressed as numbers and percentages. Statistical analysis was performed using SPSS 22.0 and a two-sided *p*-value of ≤0.05 was regarded as statistically significant in this study.

## Results

### Clinical and epidemiological features of COVID-19 patients

In total, 2188 individuals presenting with suspicious symptoms of COVID-19 were included. Twenty-four patients were diagnosed with COVID-19, with a positive rate of 1.1%. Of these patients, the average age was 46.2 years and 54.2% (13/24) were men. Fever (75.0%, 18/24) was the most frequent symptom, followed by cough (20.8%, 5/24). Abnormal findings were observed on the chest computed tomography scans in most patients (91.7%, 22/24). Besides, one COVID-19 case had coexisting HPIV infection (4.2%, 1/24), and the remaining COVID-19 patients were free from the other 12 respiratory viral pathogens.

### Profiles of other respiratory viruses among cases with suspected COVID-19

There were 226 cases out of 2188 suspected COVID-19 patients were confirmed as other respiratory viruses positive. Among them, 47.8% (108/226) were male, with an average age of 41.3 years. The overall detection rate was 10.3% (226/2188). As shown in Table 1, the most frequently detected pathogens included HRV (3.2%, 71/2188), HMPV (1.6%, 35/2188) and InfB (1.2%, 26/2188). Moreover, 38 patients were positive for the influenza virus, of which 68.4% (26/38) were co-existed with InfB infection. Twelve patients infected with InfA were classified as the subgroups 09H1N1 (41.7%, 5/12) and H3N2 (58.3%, 7/12). Of the remaining viral pathogens detected, the positive detection rates were 1.2% (26/2188) for Mp, 0.8% (17/2188) for HRSV, 0.6% (14/2188) for both HADV and HBoV, 0.6% (13/2188) for HPIV, 0.5% for HCOV (11/2188), and the rarest pathogen detected was Ch (0.4%, 9/2188). Besides, the overall detection rate of respiratory virus co-infection was 1.0% (22/2188).

**Table 1:** The characteristics of respiratory viral pathogens from suspicious COVID-19 patients

Viral pathogen	All positive	Mono virus	Two viruses	Triple or more viruses	Detection rate	Virus specific co-infection proportion
SARS-CoV-2	24	23	1	–	1.1%	4.2%
HRV	71	56	14	1	3.2%	21.1%
HMPV	35	34	1	–	1.6%	2.9%
Mp	26	25	–	1	1.2%	3.8%
InfB	26	24	2	–	1.2%	7.7%
HRSV	17	15	2	–	0.8%	11.8%
HADV	14	12	2	–	0.6%	14.3%
HBoV	14	2	11	1	0.6%	85.7%
HPIV	13	11	2	–	0.6%	15.4%
HCOV	11	6	5	–	0.5%	45.5%
Ch	9	9	–	–	0.4%	–
H3N2	7	6	1	–	0.3%	14.3%
09H1N1	5	5	–	–	0.2%	–

*Abbreviations*: *SARS-CoV-2* severe acute respiratory syndrome coronavirus 2, *HRV* human rhinovirus, *HMPV* human metapneumovirus, *Mp* mycoplasma pneumoniae, *InfB* influenza B virus, *HRSV* human respiratory syncytial virus, *HADV* human adenovirus, *HBoV* human bocavirus, *HPIV* human parainfluenza viruses, *HCOV* human coronavirus (229E, OC43, NL63, HKU1), *Ch* chlamydia. H3N2, seasonal H3N2 virus; 09H1N1, influenza A virus H1N1 (2009)

Eleven patterns of co-infection (simultaneous infection with two or more viruses) were evaluated in this surveillance research. The detailed information on these co-infection patterns is summarized in Table 2. Of the 226 cases infected with other respiratory viruses, there were 22 (9.7%) cases co-infected with other respiratory pathogens. The co-infection patterns of two or more viruses are provided in Table 2. The most dominant co-infectious agents were HBoV and HRV, accounting for 45.5% (10/22) of all co-infection cases. Moreover, it was found that 85.7% (12/14) of HBoV-positive cases had co-infections with other viruses.

**Table 2:** Co-infections types of double and triple viruses

Number	Type	co-infectious pathogens	Cases
1	HBoV +HRV	2	10
2	HCOV+HRV	2	2
3	HCOV+HMPV	2	1
4	HCOV+HPIV	2	1
5	HCOV+InfB	2	1
6	H3N2 + HRSV	2	1
7	HADV+HRV	2	1
8	HADV+ HBoV	2	1
9	HRSV+HPIV	2	1
10	HRV + InfB	2	1
11	SARS-CoV-2 + HPIV	2	1
12	HBoV +HRV + Mp	3	1

*Abbreviations*: *HBoV* human bocavirus, *HRV* human rhinovirus, *HCOV* human coronavirus (229E, OC43, NL63, HKU1), *H3N2* influenza A seasonal H3N2 virus, *HRSV* human respiratory syncytial virus, *HADV* human adenovirus, *HPIV* human parainfluenza viruses, *InfB* influenza B virus, *HMPV* human metapneumovirus, *Mp* mycoplasma pneumoniae

### Comparison of laboratory features between patients with COVID-19 and other respiratory infections

As shown in Table 3, the values of most laboratory indicators were relatively similar between patients with COVID-19 patients and those infected with other respiratory pathogens. There were no significant discrepancies between the two groups concerning age and gender distributions. The levels of triglyceride (TG) and alkaline phosphatase (ALP) were increased and decreased in COVID-19 cases, respectively, when compared to those infected with other respiratory pathogens (*p* < 0.05).

**Table 3:** Laboratory features of the COVID-19 patients and patients with other respiratory virus infections

Characteristics	Cases with COVID-19	Cases with other viral infections	*P*
Age - years	46.2 ± 17.4	41.3 ± 19.3	0.238
Gender-M(%)	13 (54.2)	108 (47.8)	0.552
Erythrocyte (× 10^12^/L)	4.7 ± 0.6	4.5 ± 0.9	0.505
Hemoglobin (g/L)	142.3 ± 18.0	135.2 ± 26.9	0.376
Hematocritc	0.4 ± 0.1	0.4 ± 0.1	0.569
Platelets (× 10^9^/L)	204.3 ± 72.0	199.3 ± 70.0	0.789
Leucocytes (×10^9^/L)	5.3 (3.7–10.2)	7.2 (5.6–9.8)	0.095
Neutrophils (%)	67.8 ± 13.9	68.3 ± 14.1	0.893
Lymphocytes (%)	22.4 ± 11.2	20.1 ± 11.3	0.438
Monocytes (%)	8.9 ± 3.3	8.3 ± 3.4	0.559
TBIL (umol/L)	8.6 (6.2–20.0)	8.8 (6.7–12.5)	0.762
DBIL (umol/L)	3.3 (2.1–6.0)	3.2 (2.2–5.0)	0.886
ALT (IU/L)	18.5 (12.0–22.8)	17.0 (12.0–30.0)	0.988
AST (IU/L)	21.0 (14.0–27.8)	22.0 (17.0–29.0)	0.399
TP(g/L)	69.9 ± 5.1	71.8 ± 10.0	0.515
ALB(g/L)	42.3 ± 3.6	41.5 ± 7.8	0.751
GLB(g/L)	27.7 ± 3.7	30.3 ± 5.9	0.134
A/G	1.6 ± 0.3	1.4 ± 0.4	0.247
GLU (mmol/L)	6.2 ± 1.5	6.7 ± 2.2	0.463
UREA (mmol/L)	4.6 (3.0–5.5)	4.1 (3.3–6.2)	0.865
CREA (umol/L)	82.5 (60.5–97.8)	72.5 (61.3–88.5)	0.579
Cys-C (mg/L)	0.9 ± 0.3	1.1 ± 0.8	0.481
URIC (umol/L)	291.5 ± 112.5	294.5 ± 95.3	0.921
TG (mmol/L)	2.2 ± 1.5	1.3 ± 0.8	0.003
CHOL (mmol/L)	3.8 ± 0.9	3.9 ± 1.1	0.762
HDL-C (mmol/L)	1.1 ± 0.3	1.1 ± 0.5	0.740
LDL-C (mmol/L)	2.3 ± 0.6	2.3 ± 0.9	0.426
ALP (IU/L)	61.5 (53.5–68.8)	82.0 (65.0–97.0)	0.002
GGT (IU/L)	21.0 (11.8–34.0)	23.0 (16.0–46.0)	0.267
CK (IU/L)	107.0 (74.8–118.0)	77.0 (51.0–116.0)	0.261
LDH (IU/L)	180.5 (154.8–212.5)	184.0 (166.3–238.5)	0.251
HBDH (IU/L)	139.5 (118.3–170.0)	139.0 (122.5–181.0)	0.607
TBA (umol/L)	2.8 (1.9–5.6)	5.0 (3.32–7.15)	0.088

For continuous variables, mean (SD) and median (IQR) were used for normally and abnormally distributed data. *TBIL* total bilirubin, *DBIL* direct bilirubin, *ALT* alanine aminotransferase, *AST* aspartate aminotransferase, *TP* total protein, *ALB* albumin, *GLB* globulin, *A/G* ratio of albumin to globulin, *GLU* glucose, *UREA* urea, *CREA* creatinine, *Cys-C* serum cystatin C, *URIC* uric acid, *TG* triglyceride, *CHOL* cholesterol, *HDL-C* high-density-lipoprotein cholesterol, *LDL-C* low-density-lipoprotein cholesterol, *ALP* alkaline phosphatase, *GGT* glutamyl transpeptidase, *CK* creatine kinase, *LDH* lactic dehydrogenase, *HBDH* hydroxybutyrate dehydrogenase, *TBA* total bile acid

## Discussion

Since the first case of COVID-19 was identified in Wuhan, China, in late December 2019, the outbreak has gradually spread nationwide, and is now rapidly spreading across many countries worldwide. In the meantime, it is also the peak season for respiratory tract infections, and their severe clinical symptoms and cross-species transmission patterns pose a huge threat to human health. Therefore, it is vital for clinicians to timely and accurately identify patients who are very likely to have COVID-19. However, it is difficult to distinguish between the patients with ARIs and COVID-19, mainly since their clinical manifestations are nearly identical at the outset. Although the etiology of ARIs is complicated, some common respiratory viruses have been reported as the leading causes of the disease [11, 12]. Hence, we should attach great importance to ARI patients infected with other viral pathogens, and at the same time, clarify the actual infection rate of SARS-CoV-2 in Southwest China. This may help us to better control the epidemic outbreak and correctly handle other respiratory tract infections.

In this study, a total of 24 patients were confirmed to have COVID-19 infection, with a positive detection rate of 1.1%. Up until 29 February 2020, 538 COVID-19 cases had been diagnosed in Sichuan province, and 143 of them were from Chengdu. However, a total of 67,773 patients were infected with COVID-19 in Hubei. Sichuan province ranked 11th in the country regarding the number of COVID-19 cases and is thus considered a mild epidemic area. The West China Hospital of Sichuan University belongs to a comprehensive tertiary hospital rather than a communicable disease hospital or designated hospital during the early outbreak period. Besides, 10.3% of the suspected COVID-19 patients were positive for other respiratory viruses. Moreover, the infection rates of common respiratory viruses were much higher compared to the SARS-CoV-2 infection. These results indicate that we should simultaneously pay close attention to ARIs patients caused by other respiratory viral pathogens, particularly in a mild epidemic area, even during the COVID-19 outbreak.

Among the 12 types of common respiratory pathogens, the most are HRV (3.2%, 71/2188), which also represents one of the most important etiological agents for respiratory tract infection. One study conducted by Kong et al. [13] showed that the positive rate of rhinovirus/enterovirus was 4.8% among 806 adult patients with ARIs in Shanghai. HMPV is a leading cause of ARIs, which exhibits a positive rate of 1.6% in our cases with suspected COVID-19. Consistently, Li et al. [14]. have reported a positive rate of 1.7% for HMPV in 2936 patients with ARIs in Beijing, China. It is worth noting that most studies have focused on the epidemiological features of HMPV in the vulnerable population. For instance, Zhang and colleagues [15] performed a cross-sectional study in Guangzhou, and reported that 103 out of 5133 pediatric patients with ARIs (2.0%) were positive for HMPV. In the present study, 38 patients were positive for influenza virus infection, of which 68.4% were InfB infection, and the remaining were InfA infection. These results indicate that InfB is the most predominant respiratory pathogen, which may differ from those reported in other studies [13, 16]. The findings of Kong et al. [13] showed that the positive rates of InfA and InfB were 40.5 and 14.4%, respectively. Another study conducted in Chongqing demonstrated a positive rate of 13.3% for influenza viruses among 24,868 patients with influenza-like illnesses (65.7% InfA, 34.1% InfB, and 0.2% co-infection of InfA and InfB co-infection) [16]. The geographical location difference, limited sample size and research duration may partly explain these contradictory findings, particularly in the etiological features of influenza viruses. Therefore, further investigation with a larger sample is urgently needed to explore in-depth the etiological features of influenza virus infection.

Previous studies have suggested the possible co-infections of multiple viruses [17, 18]. Ren et al. [18] demonstrated that viral pathogens were detected in 34.6% of ARIs samples, and 1.6% of the patients tested positive for more than one virus. Both double and triple infections were also found in this study, and the majority of HBoV-positive cases were co-infected in a combined infectious state with other pathogens. These findings are consistent with those of a previous study [19]. Despite the lack of data on this topic, co-infections of influenza virus [20], HMPV [21] and/or seasonal coronaviruses (e.g., CoV-HKU1) [22] have been reported in adults and children infected with SARS-CoV-2, which can affect the morbidity and mortality of COVID-19. To the best of our knowledge, this study was the first to show that one COVID-19 case had concurrent infections with HPIV. These data on the co-infection of SARS-CoV-2 with common respiratory viruses or non-SARS-CoV-2 respiratory viral pathogens imply that the routine testing of multiple viral pathogens should not be ignored in mild epidemic areas, even during the COVID-19 outbreak. However, whether simultaneous viral infection in SARS-CoV-2 patients can potentially drive viral interference or impact disease outcome awaits further research.

In terms of laboratory features, most of the blood biochemical indicators were relatively similar between the patients with COVID-19 infection and those with other respiratory tract infections, except that the levels of TG and ALP were higher and lower respectively in COVID-19 cases. These results suggest that SARS-CoV-2 infection exhibits similar features with many other respiratory virus infections. However, a recent study on the comparison of the clinical features between COVID-19 patients and non-SARS-CoV-2 virus-infected pneumonia cases demonstrated that COVID-19 patients had higher levels of aspartate aminotransferase, alanine aminotransferase, lactate dehydrogenase, γ-glutamyl transpeptidase and α-hydroxybutyric dehydrogenase [23]. Thus, additional evidence is still required for the mechanism of higher TG but lower ALP levels in COVID-19 patients and the overall analysis of their clinical features.

## Conclusion

This study analyzed the epidemiological patterns of different respiratory viruses, and found that although the widespread of COVID-19 is a serious public health concern, SARS-CoV-2 may not be the main pathogen responsible for respiratory tract infection in Southwest China, a mild epidemic area. Therefore, effective surveillance of the transmission patterns of respiratory tract pathogens in the COVID-19 affected area may be conducive for us to formulate an optimal treatment regimen and inhibit the rapid spread of the virus.

## Data Availability

The data and materials supporting the conclusions of the study are available from the corresponding author on reasonable request.

## Abbreviations

COVID-19: Coronavirus disease 2019
SARS-CoV-2: Severe acute respiratory syndrome coronavirus 2
ARIs: Acute respiratory infections
09H1N1: Influenza A (H1N1) 2009 virus
H3N2: Seasonal H3N2 virus
InfB: Influenza B virus
HADV: Human adenovirus
HBoV: Human bocavirus
HRV: Human rhinovirus
HPIV: Human parainfluenza viruses
HCOV: Human coronavirus (229E, OC43, NL63, HKU1)
HRSV: Human respiratory syncytial virus
HMPV: Human metapneumovirus
Mp: Mycoplasma pneumonia
Ch: Chlamydia
